# Cancer-related fatigue stratification system based on patient-reported outcomes and objective outcomes: A cancer-related fatigue ambulatory index

**DOI:** 10.1371/journal.pone.0215662

**Published:** 2019-04-22

**Authors:** Antonio Cuesta-Vargas, Jena Buchan, Bella Pajares, Emilio Alba, Cristina Roldan-Jiménez

**Affiliations:** 1 Department of Physiotherapy, Faculty of Health Sciences, Universidad de Malaga, Málaga, Spain; 2 Institute of Biomedical Research in Málaga, Málaga, Spain; 3 School of Clinical Science, Faculty of Health Science, Queensland University Technology, Brisbane, Australia; 4 School of Allied Health Sciences, Griffith University, Gold Coast, Australia; Iranian Institute for Health Sciences Research, ISLAMIC REPUBLIC OF IRAN

## Abstract

Although breast cancer mortality is decreasing, morbidity following treatment remains a significant issue, as patients face symptoms such as cancer-related fatigue (CRF). The aim of the present study is to develop a classification system that monitors fatigue via integration of an objective clinical assessment with patient self-report. Forty-three women participated in this research. Participants were post-treatment breast cancer survivors who had been surgically treated for their primary tumour with no evidence of neoplastic disease at the time of recruitment. Self-perceived fatigue was assessed with the Spanish version of the Piper Fatigue Scale-Revised (R-PFS). Objective fatigue was assessed by the 30 second Sit-to-Stand (30-STS) test. Confirmatory factor analysis was done with Maximum Likelihood Extraction (MLE). Internal consistency was obtained by Cronbach's α coefficients. Bivariate correlation showed that 30-STS performance was negatively-inversely associated with R-PFS. The MANOVA model explained 54.3% of 30-STS performance variance. Using normalized scores from the MLE, a classification system was developed based on the quartiles. This study integrated objective and subjective measures of fatigue to better allow classification of patient CRF experience. Results allowed development of a classification index to classify CRF severity in breast cancer survivors using the relationship between 30-STS and R-PFS scores. Future research must consider the patient-perceived and clinically measurable components of CRF to better understand this multidimensional issue.

## Introduction

While breast cancer is the most commonly diagnosed type of cancer in women, accounting for 30% of all new diagnoses, mortality rates have decreased by 38% in recent years [1]. However, breast cancer still represents 15% of all cancer-related deaths in women [2]. Despite decreasing mortality rates, morbidity following breast cancer remains a significant issue in the growing group of survivors. Survivorship starts at the time of cancer diagnosis and lasts throughout the lifespan, and it is commonly accompanied by treatment-related side effects [3]. Cancer-related fatigue (CRF) is a symptom reported in about 70% of patients suffering from cancer, both during and shortly after treatment. Additionally, up to 30% of survivors develop CRF years post-treatment [4]. However, these rates may be even higher given the difficulty in diagnosing and assessing CRF. For example, research has reported from 56% to 95% of breast cancer survivors experience CRF post-treatment [5].

Experience of CRF is essential to monitor for, to enable healthcare practitioners to provide treatment and monitor its effectiveness [6]. Furthermore, the presence of CRF is typically accompanied by financial implications, including an increased utilization of health care resources [7], and decreased capability to work or return to work [8]. Also, CRF is associated with higher levels of depression, pain and sleep disturbance, commonly leading to a reduction in the quality of life [9]. In addition, the effect of exercise may vary depending on fatigue baseline levels, so its assessment allows targeting specific subgroups for better benefits and cost effectiveness [10].

Fatigue has been defined as a multidimensional concept involving psychological and physiological dimensions [11]. Given this, there is a needto assess it in a subjective and an objective manner [12], but the complexity makes it a challenging variable to measure with a single tool [13]. In the oncology field, CRF is defined as a feeling of low energy, weariness or tiredness, and is characterized based on its severity, distress and inability to find relief with rest [4]. The aetiology of CRF remains to be fully elucidated and is considered multifactorial, including side-effects of treatment and psychological factors [14]. In fact, CRF is the most variable symptom during and post-treatment [15], highlighting the need for better assessment and monitoring techniques, as well as management options.

In self-reported fatigue, patient-reported outcomes (PRO) are the primary assessment tool. PROs are used to assess a patient's symptoms or functional status at a specific time [16,17]. Although PRO data are subjective, they can help understand how a condition or disease influences a patient's capabilities, as well as detect self-perceived changes due to an intervention [18]. Hence, several questionnaires have been developed to asses CRF, such as the Quick-Piper [19] and Piper Fatigue Scale-revised (R-PFS) [20]. More recently, a systematic review reported a core set of measures to asses CRF, all of which were PROs [6].

While PRO tools offer ease in self-monitoring and assessment [15] and are widely used in the current literature [6], the aforementioned physical and psychological complexity of CRF suggests an objective measure is needed to complement the subjective measure of CRF. In exercise physiology, objective changes in task performance provide information about performance fatigability, expressed as the capacity of a muscle or muscle group to generate force, and it is part of neuromuscular function assessment [21]. In the oncology field, the neuromuscular domain of fatigue has been less studied. In this respect, limited physiological measures of CRF have been assessed to measure its central and peripheral domains, with findings that are of limited functional relevance [22–24]. A potential assessment tool to compliment PRO measures of fatigue is the 30 second sit-to-stand (30-STS) test [25]. By assessing number of repetitions completed, it has been used in the evaluation of functional fitness levels [26] and in rehabilitation [27], including use in the oncology setting to measure lower extremity endurance in prostate cancer [28], head and neck cancer [29] and breast cancer survivors [30,31].

Given the high prevalence, variability and multidimensionality of CRF in breast cancer survivors, work is needed to develop a more complete objective and subjective assessment of CRF. Hence, the aim of the present study is to develop a classification system that improves clinical assessment and monitoring of fatigue via integration of an objective clinical assessment with patient self-report, using the 30-STS and R-PFS, respectively.

## Materials and methods

### Subjects

Forty-three women aged between 32 and 69 years old volunteered to participate in this cross-sectional study. They were recruited by Medical Oncology faculty specialists from the University Clinical Hospital Virgen de la Victoria (Málaga, Spain). Participants were post-surgery breast cancer survivors who had been surgically treated for their primary tumour with no evidence of neoplastic disease at the time of recruitment. Patients undergoing hormonal treatment were allowed to participate. Exclusion criteria included suffering any cardiovascular event defined as: stable or unstable angina; acute pulmonary oedema; cardiac rhythm disorders; and syncope of cause not affiliated in the year prior to inclusion. All participants signed an informed consent prior to participation. Ethical clearance for the study, following the Helsinki declaration, was obtained from Ethics Committee of the Provincial Research of Málaga, Andalusian Health Service (approval number 2804/2016).

### Procedure

Participants completed testing at the hospital. In addition to providing demographic and clinical history, they completed the following assessments:

- Self-perceived fatigue: The Spanish version of the Piper Fatigue Scale-Revised (R-PFS) was used to obtain a subjective PRO of fatigue. This self-administered questionnaire contains 22 items whose scores range from 0 to 10 and includes four domains of subjective fatigue: behavioral/severity (6 items); affective meaning (5 items); sensory (5 items); and cognitive/mood (6 items). The scale has high reliability (Cronbach’s α = 0.96) in the breast cancer population [20]. Further, this Spanish version of the scale has been validated in Spanish breast cancer survivors, showing satisfactory psychometric properties [32]. This questionnaire was completed during the session, with the researcher available to clarify questions when required.
- Clinical assessment of fatigue: To obtain an objective, clinical assessment of fatigue, the 30 second Sit-to-Stand (30-STS) test was used. This test has been demonstrated to produce quadriceps fatigue [25], used to assess variables such as lower body endurance in cancer survivors [30]. The 30-STS was selected as it is a highly functional and transferable test, providing a quantitative measure of an important activity of daily living essential for independence [25]. To perform the test, the subject has to sit and rise from a 43-centimetre-high chair for 30 seconds, moving as rapidly as possible through the entire range of motion. The task begins in the standing position, with feet hip-width apart and upper limbs crossed over the anterior of the body to avoid impulses [25].

### Data analysis

Descriptive analyses were used to present mean, standard deviation, minimum and maximum of anthropometric variables. Distribution and normality of variables were determined using one-sample Kolmogorov-Smirnov tests. Association between 30-STS performance and R-PFS scores, overall and for each domain, was reported using Pearson´s Correlation Coefficient (r). A multivariate analysis of variance (MANOVA) was completed to further examine relationships between 30-STS performance (number of repetitions) and both overall and individual domain scores on the R-PFS. A confirmatory factor analysis with Maximum Likelihood Extraction (MLE) was employed to determine if sample size was sufficient to construct a classification system with standard scores [33]. Internal consistency was obtained by Cronbach's α coefficients at an anticipated value range of 0.80–0.95 [34,35]. A two-sided 5% significance value (p) was used for all analyses, performed using SPSS 22.0 for Windows.

## Results

Participants had undergone a lumpectomy (60%) or mastectomy (40%). Further, 93.3% of them had been treated with chemotherapy, radiotherapy and/or hormone therapy, with the vast majority (75%) still on hormone therapy. Additional demographic and clinical variables are presented in Table 1.

**Table 1:** Participant demographic and clinical variables.

	Mean±SD	Min-Max
**Age (years)**	51.6±8.9	32.0–69.0
**BMI (Kg/m**^**2**^**)**	28.5±5.0	20.3–42.0
**Years from diagnosis**	2.0±1.7	0–8.0
**Surgical Intervention**		**Percentage (n)**
	Lumpectomy	60.0% (26)
	Mastectomy	40.0% (17)
**Cancer Treatment**		
	Chemotherapy	93.3% (40)
	Radiotherapy	93.3% (40)
	Hormone Therapy	93.3% (40)
	Monoclonal Antibody	31.1% (13)
**Current treatment**		
	None	13.6% (7)
	Radiotherapy	6.8% (3)
	Monoclonal antibody	4.5% (2)
	Hormone therapy	75.0% (32)

Bivariate correlation showed that 30-STS test was negatively-inversely associated with Total Piper Score and all its domains (Table 2). However, this correlation was not significant in Domain III (p = 0.089).

**Table 2:** Pearson correlation (r,p) between 30-STS test and R-PFS.

Total R-PFS score	Domain I	Domain II	Domain III	Domain IV
-0.379 (0.010)	-0.482 (0.001)	-0.461 (0.002)	-0.259 (0.089)	-0.438 (0.003)

Domain I = behavioral/severity; Domain II = sensory/mood; Domain III = Cognitive; Domain IV = affective/meaning

Total R-PFS and individual domain scores were used to develop a MANOVA model examining contribution of total and individual domain scores to 30-STS results. This model explained 54.3% of 30-STS result variance. Based on the decomposition of the model, the main contributing variable was Domain III (p = 0.247) followed by Domain II (p = 0.251). However, these variables did not reach the required value to explain the model by themselves (Table 3).

**Table 3:** Decomposition of the multivariate model.

**Model**		**R**		**R Square**		**Adjusted R Square**		**Std. Error of the Estimate**	
1		0.543		0.295		0.200		4,80950	
			**Unstandardized Coefficients**				**Standardized Coefficients**	t	*p*
			B		Std. Error		Beta		
**(Constant)**		24.181		1.963			12.320	0.000
**Total Piper**			-.026		0.036		0-.241	-0.725	0.473
**DOMAIN I**			-.125		0.143		-0.219	-0.874	0.388
**DOMAIN II**			-.134		0.115		-0.250	-1.166	0.251
**DOMAIN III**			.202		0.172		0.302	1.176	0.247
**DOMAIN IV**			-.104		0.183		-0.132	-0.569	0.572

Domain I = behavioral/severity; Domain II = sensory/mood; Domain III = Cognitive; Domain IV = affective/meaning

Kaiser–Meyer–Oklin values (0.865) and the Bartlett’s test of sphericity (Chi-squared value = 173.42 and gl 15) (p<0.001) indicated the correlation matrix was adequate for the Maximum Likelihood Extraction. Maximum Likelihood Extraction detected one factor with Eigenvalues above 1, explaining 69.17% of the total variance (See S1 Appendix). Using these normalized scores, a classification system was developed based on the quartiles (See S2 Appendix). This allowed CRF level classification of each participant, based on the relationship of 30-STS performance and R-PFS score (Table 4).

**Table 4:** Cancer-related fatigue classification scale.

CRF level	Normalized ranges
**Subclinical**	< -0.70
**Mild**	-0.70 –-0.22
**Moderate**	-0.22–0.31
**Severe**	0.31–0.80
**Extreme**	> 0.80

## Discussion

This study attempted to integrate objective and subjective assessments of fatigue,allowing more thorough classification of patient experience. Study aims were achieved, as results allowed development of an ambulatory index to assess CRF in breast cancer survivors. Results of the 30-STS were correlated with scores from total and each domain of the R-PFS to examine associations and relationships. Based on an observed relationship between the objective (30-STS) and subjective (R-PFS) tests, a scale was developed classifying CRF as one of five levels: Subclinical, mild, moderate, severe or extreme. As highlighted, CRF is multidimensional and includes physical and psychosocial components, necessitating an assessment that captures all domains. While PRO outcome tools such as the R-PFS [19] and Quick-Piper [20] are easy to access and provide an idea of how CRF affects the individual, combining an objective measure allows further assessment of how someone’s daily functional ability may also be impacted. Extensive research highlights the negative impact CRF has on quality of life through its impact on daily functioning ability [36–38].

Results from the Pearson correlations highlighted that 30-STS performance was inversely associated with both total and individual domain scores of the R-PFS. As expected, individuals with higher self-perceived fatigue demonstrated poorer performance on the objective test. That is to say, a higher score on R-PFS (suggesting greater perceived fatigue), the fewer repetitions achieved in the 30-STS test. Of note is that the association between 30-STS performance and the cognitive domain of fatigue (Domain III) did not reach statistical significance. This domain relates to fatigue contributing to things like decreased attention span and impaired perception, sometimes considered to be ‘attention fatigue’ [39]. Given the relatively small directed attention demands of the study, it is feasible fatigue in this domain was not overly affecting at the time.

Based on MANOVA results, 54.3% of 30-STS result variance was explained by R-PFS scores. Hence, the number of repetitions is explained 54.3% by self-perceived fatigue, while the remaining 45.7% of variability in the number of repetitions is explained by other factors. Future research could look to further examine other key variables associated with both 30-STS results and R-PFS. As breast cancer survivors with CRF often experience a range of other physical and psychosocial side effects [40], including decreased social interaction, mood, cognitive functioning and work performance, there may be other objective and subjective measures closely linked with self-reported fatigue. Given the multiple dimensions of fatigue, as highlighted by different scores for each R-PFS domain and varying performance in the 30-STS, it is unlikely a single test can adequately monitor CRF.

Based on the analyses and results, a classification system was devised using the observed relationship between 30-STS and R-PFS scores (Table 4). Five rankings were developed, ranging from subclinical to extreme, resulting in a fatigue severity scale incorporating objective and subjective aspects of the condition. A single score would support both clinician and patient in monitoring fatigue with an ambulatory index. This way, classification incorporates a subjective, self-reported measure and an objective measure like the 30-STS.

Previous longitudinal studies have subjectively monitored the trajectory of CRF throughout breast cancer treatment. However, the focus has spanned from mean levels of fatigue to individual levels, making it difficult to get a full idea of overall experience. These studies have resulted in both differentiation of subgroups suffering from high versus low levels of fatigue [41,42], as well as one study involving a daily assessment of fatigue [43]. An addition study sought to develop a broader, more transferable classification scale similar to the current research, distinguishing between five levels of fatigue: High, Recovery, Late, Low, and Very Low [44]. However, all mentioned studies only assessed and developed classification systems using subjective measures of CRF via PRO. As previously highlighted, given fatigue is a multi-dimensional experience with physical and psychological components, inclusion of an objective parameter has strong clinical relevance to better understand the patient experience and prescribe more encompassing, individualized treatment. Specifically, understanding CRF experience from a more physical side, such as via a 30-STS, would allow better prescription of exercise. This is of significant clinical relevance, considering that exercise has been demonstrated as more effective for CRF management than pharmaceutical and psychological treatments [45], and that a patient-specific level of moderate intensity is recommended for key effectiveness [46]. Hence, this classification system, drawing on subjective and objective measures, would allow more individualized treatment prescription, including exercise, as well as understanding of CRF experience severity to ensure more appropriate, tailored allocation of treatment resources and focus.

This approach to patient classification is used in other groups outside of cancer survivors. For example, the CODI index is an on-line classification tool used to rate severity of fibromyalgia symptoms [47]. Clinicians and researchers can easily access this tool to assess symptom severity and prescribe treatment or assess the effect of an intervention quickly and more objectively than traditional means. The classification system developed in this study may serve a similar purpose, whereby clinicians can use results from two quick, easily accessible tests (30-STS and R-PFS) to obtain a more complete picture of their patient’s CRF and treat accordingly. For example, an individual displaying subclinical levels of fatigue may be able to undergo fewer follow-ups and have the focus on more pressing issues, whereas those in the ‘severe’ or ‘extreme’ categories may benefit from greater attention to their fatigue over other issues. This is supported by substantial research highlighting the impact CRF has on all dimensions of life, reported by cancer survivors as one of the most distressing symptoms during and following cancer [38–40,48].

Limitations of this research must be acknowledged. These include a relatively small sample size, only post-treatment survivors and lack of assessment related to the influence of other clinical, psychosocial and socio-demographic factors on results. Further research should look to examine if the association between 30-STS performance and R-PFS remains similar in women still undergoing breast cancer treatment, as well as in other types of cancer. There is also a need to examine what other variables may impact 30-STS performance and correlation with self-reported fatigue levels, as well as greater understanding of the influence of the four fatigue dimensions. Study strengths included range in participant age, years since diagnosis and type of surgery undergone, as more extensive surgery, particularly when involved axillary lymph node dissection, has been linked to greater experience of CRF-related problems [37].

In summary, the fatigue classification system developed in this study has promising clinical and research implications. It supports a more well-rounded, but easily accessible, assessment of CRF and treatment evaluation. By combining objective and subjective measures of fatigue and using these to classify the severity of an individual’s experience of the condition, management strategies can be better prescribed. Additionally, those conducting research on interventions to help with the management and treatment of CRF may use the scale for easier assessment of intervention effectiveness. Patients may also use the tool to track their fatigue and better understand their contributing factors and more effective self-management techniques. As highlighted, future research must consider the patient-perceived and clinically measurable components of CRF to better understand this multidimensional issue.

## Conclusions

This study integrated objective and subjective assessments of fatigue to better allow classification of patient experience. Results allowed development of an ambulatory index to assess CRF in breast cancer survivors using the relationship between 30-STS and R-PFS scores. By combining objective and subjective measures of fatigue and using these to classify the severity of an individual’s experience of the condition, management strategies can be better prescribed. Future research must consider the patient-perceived and clinically measurable components of CRF to better understand this multidimensional issue.

## Supporting information

S1 Appendix Confirmatory factor analysis with maximum likelihood extraction. (DOCX)

S2 Appendix Cancer-related fatigue (CRF) index: Normalized scores. (DOCX)

S1 Database CLUB DE SALUD_ingles(1)_plosone(1). (SAV)
