# Supplementary material for: Cancer-related fatigue stratification system based on patient-reported outcomes and objective outcomes: A cancer-related fatigue ambulatory index
Source: PLoS One. 2019 Apr 22;14(4):e0215662. doi: 10.1371/journal.pone.0215662 (PMC6476532; doi:10.1371/journal.pone.0215662)
Supplement: S1 Appendix — (DOCX) [file pone.0215662.s001.docx]

**S1 Appendix. Confirmatory Factor Analysis with Maximum Likelihood Extraction.**

**S1.1.** Kaiser-Meyer-Olkin and Bartlett's Test of Sphericity.

| **KMO and Bartlett's Test** | | |
| --- | --- | --- |
| Kaiser-Meyer-Olkin Measure of Sampling Adequacy. | | ,865 |
| Bartlett's Test of Sphericity | Approx. Chi-Square | 173,422 |
|  | df | 15 |
|  | Sig. | ,000 |

**S1.2.** Communalities.

| **Communalities** | | |
| --- | --- | --- |
|  | Initial | Extraction |
| 30-STS | ,295 | ,232 |
| Sum of score of all items (TOTAL PIPER) | ,831 | ,899 |
| DOMAIN I  Behavioral/severity | ,703 | ,713 |
| DOMAIN II sensory/mood | ,601 | ,584 |
| DOMAIN III Cognitive | ,721 | ,719 |
| DOMAIN IV Affective/meaning | ,649 | ,677 |
| Extraction Method: Maximum Likelihood. | | |

**S1.3.** Total Variance Explained

| **Total Variance Explained** | | | | | | |
| --- | --- | --- | --- | --- | --- | --- |
| Factor | Initial Eigenvalues | | | Extraction Sums of Squared Loadings | | |
|  | Total | % of Variance | Cumulative % | Total | % of Variance | Cumulative % |
| 1 | 4,150 | 69,169 | 69,169 | 3,824 | 63,732 | 63,732 |
| 2 | ,766 | 12,764 | 81,932 |  |  |  |
| 3 | ,391 | 6,514 | 88,446 |  |  |  |
| 4 | ,327 | 5,453 | 93,900 |  |  |  |
| 5 | ,247 | 4,119 | 98,019 |  |  |  |
| 6 | ,119 | 1,981 | 100,000 |  |  |  |
| Extraction Method: Maximum Likelihood. | | | | | | |

*Maximum Likelihood Extraction detected one factor with Eigenvalues above 1, explaining 69.17% of the total variance*

S1.4. **Factor Matrix**

| **Factor Matrix^a^** | |
| --- | --- |
|  | Factor |
|  | 1 |
| 30-STS | -,482 |
| Sum of score of all items (TOTAL PIPER) | ,948 |
| DOMAIN I  Behavioral/severity | ,845 |
| DOMAIN II sensory/mood | ,764 |
| DOMAIN III Cognitive | ,848 |
| DOMAIN IV Affective/meaning | ,823 |
| Extraction Method: Maximum Likelihood. | |
| a. 1 factors extracted. 5 iterations required. | |

| **Estadísticos** | | |
| --- | --- | --- |
| REGR factor score 1 for analysis 1 |  |  |
| N | Válido | 43 |
|  | Perdidos | 12 |
| Percentiles | 20 | -,7048164 |
|  | 25 | -,5853474 |
|  | 40 | -,2245673 |
|  | 50 | ,1971410 |
|  | 60 | ,3188371 |
|  | 75 | ,5167998 |
|  | 80 | ,8087109 |
