# Supplementary material for: Cancer-related fatigue stratification system based on patient-reported outcomes and objective outcomes: A cancer-related fatigue ambulatory index
Source: PLoS One. 2019 Apr 22;14(4):e0215662. doi: 10.1371/journal.pone.0215662 (PMC6476532; doi:10.1371/journal.pone.0215662)
Supplement: S2 Appendix — (DOCX) [file pone.0215662.s002.docx]

**S2 Appendix. Cancer-related fatigue (CRF) index: normalized scores**

**S2.1.** Normalized scores descriptive statistics

| Descriptive statistics | | | | | | | |
| --- | --- | --- | --- | --- | --- | --- | --- |
|  | N | Range | Minimum | Maximum | Mean | Standard Desviation | Variance |
| REGR factor score 1 for analysis 1 | 43 | 4,05845 | -2,04290 | 2,01555 | ,0000000 | ,97296297 | ,947 |
| N valid | 43 |  |  |  |  |  |  |

**S2.2.** Percents and quartiles for classification scale (Table 4)

| **Estatistics** | | |
| --- | --- | --- |
| REGR factor score 1 for analysis 1 |  |  |
| N | Valid | 43 |
|  | Missing | 0 |
| Percents | 20 | -,7048164 |
|  | 25 | -,5853474 |
|  | 40 | -,2245673 |
|  | 50 | ,1971410 |
|  | 60 | ,3188371 |
|  | 75 | ,5167998 |
|  | 80 | ,8087109 |

*This allowed CRF level classification of each participant, based on the relationship of 30-STS performance and R-PFS score:*

- *Subclinical: Under percentile 20 (first quartile)*
- *Mild: Between percentile 20 and 40 (second quartile)*
- *Moderate: Between percentile 20 and 40 (Third quartile)*
- *Severe: Above percentile 80 (Fifth quartile)*
- *Extreme: Between percentile 60 and 80*
